# Reporting Guidelines for Community-Based Participatory Research Did Not Improve the Reporting Quality of Published Studies: A Systematic Review of Studies on Smoking Cessation

**DOI:** 10.3390/ijerph17113898

**Published:** 2020-05-31

**Authors:** Daisuke Kato, Yuki Kataoka, Erfen Gustiawan Suwangto, Makoto Kaneko, Hideki Wakabayashi, Daisuke Son, Ichiro Kawachi

**Affiliations:** 1 Department of Family Medicine, Mie University Graduate School of Medicine, Mie 514-0104, Japan; 2 Hospital Care Research Unit and Department of Respiratory Medicine, Hyogo Prefectural Amagasaki General Medical Center, Hyogo 660-8550, Japan; youkiti@gmail.com; 3 Institute for Networking Development of Primary Care Clinics, Department of Medical Ethics, Law, and Primary Care, Atma Jaya Catholic University of Indonesia, Jakarta 12930, Indonesia; erfen_gs@yahoo.co.id; 4 Department of Family and Community Medicine, Hamamatsu University School of Medicine, Hamamatsu, Shizuoka 431-3125, Japan; makotokaneko0314@gmail.com; 5 Primary Care Research Unit, Graduate School of Health Data Science, Yokohama City University Yokohama, Kanagawa 236-0027, Japan; 6 Department of Community Medicine, Kameyama, Mie University School of Medicine, Mie 514-0104, Japan; hidekiw927@gmail.com; 7 Department of Medical Education Studies, International Research Center for Medical Education, Graduate School of Medicine, The University of Tokyo, Tokyo 113-8654, Japan; sondtky@gmail.com; 8 Department of Social and Behavioral Sciences, Harvard School of Public Health, Cambridge, MA 02115, USA; ikawachi@hsph.harvard.edu

**Keywords:** community-based participatory research, smoking cessation, reporting guideline, literature review

## Abstract

The objective of this study was to assess the impact of a 2010 community-based participatory research (CBPR) reporting guideline on the quality of reporting a CBPR on smoking cessation. We searched the MEDLINE, Embase, the Cochrane Central Register for Controlled Trials (CENTRAL), PsycINFO, and Cumulative Index to Nursing and Allied Health Literature (CINAHL) databases and included articles published up to December 2019 (PROSPERO: CRD42019111668). We assessed reporting quality using the 13-item checklist. Of the 80 articles identified, 42 (53%) were published after 2010. The overall reporting quality before and after 2010 was poor and did not differ significantly (mean difference: 0.66, 95% confidence interval [CI]: −0.21 to 1.53). The total reporting scores of the studies did not differ significantly according to the effect size of the intervention (beta coefficient: −2.86, 95% CI: −5.77 to 0.04). This study demonstrates the need to improve the quality of reporting CBPRs. We recommend that journal editors endorse the CBPR reporting guideline to encourage its use by more researchers.

## 1. Introduction

Reporting guidelines have played an important role in improving the quality of reporting the results of studies published in peer-reviewed scientific journals. For example, many journals have endorsed the Preferred Reporting Items for Systematic Reviews and Meta-analyses (PRISMA) Statement [1] and require that it be used for reporting the results of systematic reviews and meta-analyses [2]. This requirement has been shown to have improved the quality of reporting the results of systematic reviews and meta-analyses [3,4]. Similarly, the use of the Consolidated Standards of Reporting Trial (CONSORT) Statement [5] has improved the quality of reporting the results of randomized controlled trials (RCTs) [6,7], and this improvement has been attributed to journal endorsement of the CONSORT Statement [8].

Community-based participatory research (CBPR), a partnership approach involving collaboration with community members and researchers in the planning, implementation, and evaluation of interventions to improve health, has been proposed as a solution to community health issues [9,10]. However, it is particularly challenging to apply uniform reporting criteria to studies with a CBPR design because each community is unique, and CBPR frequently involves multifaceted interventions at multiple levels (individuals, organizations, institutions). Nevertheless, it is important to standardize the reporting criteria as much as possible to obtain generalizable knowledge [11]. A guideline for reporting the results of CBPR (“best practices”) was published in 2010 [11], but the impact of this guideline on the reporting quality of CBPR has not been evaluated. This reporting guideline is registered with the Enhancing the QUALITY and Transparency Of health Research (EQUATOR) Network (https://www.equator-network.org/), and uses a checklist approach to guide the researcher on uniform standards for reporting the results of CBPR. The checklist includes a total of 13 items, consisting of four parts: organizational structure, key elements of the project, co-researchers’ experiences, and challenges of the project.

Several studies have found that CBPR can be effective at achieving smoking cessation [12]. Tobacco smoking is a pressing public health challenge on a global scale [13], which accounts for 10% of total global deaths [14]. The total economic cost of smoking-attributable diseases has been estimated at 1.8% of the global gross domestic product [15].

We conducted this study to determine the effect of the 2010 CBPR guideline [11] on the quality of reporting the results of studies on CBPR for smoking cessation. We found that the overall reporting quality was low and did not improve after the publication of the reporting guideline.

## 2. Materials and Methods

### 2.1. Types of Articles Included

We included articles that reported the results of quantitative CBPR focused on smoking cessation, published before December 2019. We excluded articles that did not evaluate smoking cessation as an outcome such as articles on the cost-effectiveness or participant satisfaction with the intervention. We also excluded articles in which the results were abridged or republished.

CBPR has been suggested as an effective approach for the solution of many community health issues. [9,10] However, due to the length of time and investment of effort required to conduct CBPR, there is a dearth of studies targeting chronic diseases. On the other hand, CBPR interventions to promote smoking cessation can be launched in a relatively shorter time period, and accordingly, we found more studies that focused on this topic that allowed us to fulfill our study objective, viz. to evaluate the reporting quality of CBPR in published studies.

### 2.2. Search Strategy

We searched MEDLINE via PubMed, Embase via ProQuest, CENTRAL, PsycINFO, and CINAHL via EBSCOhost for articles written in English and published before December 2019. The search terms were selected to identify studies describing the results of CBPR and included terms such as “community-based participatory research”, “community-based research”, “participatory research”, or “action research”, combined with “smoking cessation” or “tobacco.” Details of the search terms are provided in Appendix S1.

This article is a review of published articles, so ethics approval and consent did not apply. We used a prespecified protocol to conduct this review. The protocol was registered in the International Prospective Register of Systematic Reviews (PROSPERO) (CRD42019111668).

### 2.3. Study Selection

Two authors screened the titles and abstracts of the articles identified in the initial search independently and decided whether each article was eligible for inclusion in the full-text review. Differences in the reviewers’ opinions were resolved by discussion.

### 2.4. Definition of Community-Based Participatory Research (CBPR)

We defined CBPR as participatory action research conducted in a community [10]. According to Minkler (2004, p. 685) [16], “CBPR is not a method per se, but an orientation to research”; therefore, the definition of CBPR can vary. More recently, some articles have clarified the concept of CBPR as involving a partnership between the researchers and the community [17,18].

We adopted the concept of CBPR to include the “community as the setting” or the “community as the target” of the intervention [19]. If we found the word “community-based” in the title/abstract/main text of an article, we included the article. In addition, if we found the word “community” and related words in the title/abstract/main text of an article, we included the article if the meaning of the word was consistent with the context of participatory action research in a community. Examples of community-related words included “families”, “social networks”, “schools”, “neighborhoods”, “churches”, “worksites,” “voluntary agencies,” “online communities”, and “other organizations”.

### 2.5. Data Extraction and Assessment of Reporting Quality

The selected papers were divided into two sets, and three authors were organized into two pairs: (1) author 1 and author 2, and (2) author 1 and author 3. The two reviewers in each pair extracted the information from each article independently. Each reviewer checked the study characteristics: area where the article had been published or the research had taken place, objectives, evaluation type (whether or not it aimed to evaluate a project for smoking cessation), study design (individual-/cluster-RCT, or quasi-experimental study), effect size, journal impact factor in 2017, the duration of the project, and the number of professionals who had participated in the research. The effect size was calculated as the risk difference between the intervention group and the control group for RCTs, and as the pre-intervention smoking rate minus the post-intervention smoking rate for studies with a before-and-after design. We included the evaluation type because we expected that studies evaluating the project for smoking cessation would have a higher reporting quality.

The reporting quality was assessed using the 14-item checklist (Appendix S2) from the guideline for reporting the results of CBPR published in October 2010 [11]. We excluded the last item on the checklist, “What can we learn?” in the domain “Address the Challenges, Pitfalls, and Limitations of the Project” because we reasoned that for a paper to be published, a reviewer must have considered it to be worth reading, and so, in principle there is something to be learnt from every published paper. The exclusion of this item reduced the number of items on the checklist to 13.

To calibrate the assessment and scoring of THE study quality, two reviewers extracted the data from five articles and evaluated them using the 2010 reporting guidelines (see Appendix S3) and compared their assessments prior to starting the main review. Any disagreements between the reviewers were resolved through discussion and, if that failed, through arbitration by a third reviewer.

### 2.6. Data Analysis

We summarized the characteristics of each study using descriptive statistics. We assessed the association between the total reporting scores according to whether the article was published before or after 2010, using Fisher’s exact test. The total number of the checklist items in the CBPR reporting guideline that had been satisfied was used as the total reporting score (range: 0–13). We evaluated the relationship between the total reporting score and study characteristics using multivariable linear regression. The analyses took into account the timing of the publication (pre-/post-guideline), journal impact factor, effect size, evaluation type, and the study design. All the analyses were performed by R Version 3.5.1 (R Foundation for Statistical Computing, Vienna, Austria).

## 3. Results

### 3.1. Search Results and Characteristics of the Articles Included

**Figure 1:**
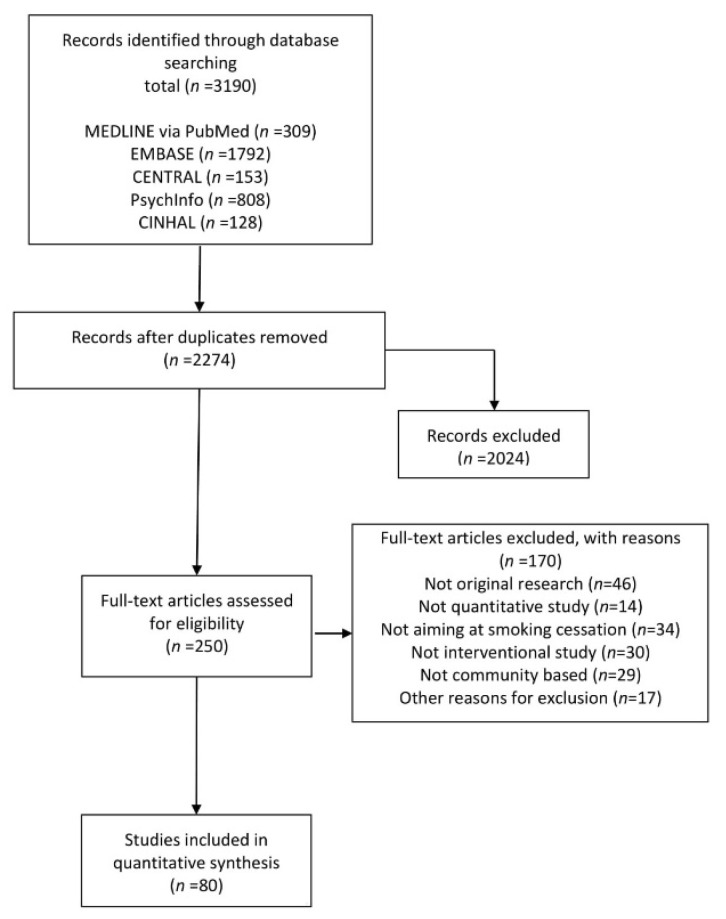
Flow chart showing the data search and study selection process.

Through the screening of titles and abstracts, the search yielded 3190 potential articles. Figure 1 provides a flow chart of the article selection process. We evaluated the full text of 250 articles, and as a result, 80 articles (published between 1987 and 2019) were included in the final review (Table S4). Of the articles selected, 42 (53%) were published after October 2010, when the reporting guideline was published.

Table S5 shows the characteristics of the studies that were included in the review. Comparing the characteristics before and after the publication of the reporting guideline, the proportion of articles that reported on an evaluation of a smoking cessation program decreased from 66% to 45%, while that of articles that reported the results of individually randomized controlled trials increased from 18% to 29%.

### 3.2. Conformity with the CBPR Reporting Guidelines

Among individual items on the 13-item checklist, the quality score ranged from 19% for item q3.1 (“Pay attention to who is writing the article and how their voices and experiences are represented”) to 100% for items q1 (article structure), q2.5 (methodology/process of the project), and q2.6 (project outcomes/emergent actions). Table 1 shows the frequency of each reporting item. Of the 13 items assessed, only one item, item 2.8 (graphics of the project design), improved significantly after the guideline was published.

### 3.3. Factors Associated with the Total Reporting Score

The total reporting score did not improve significantly after the publication of the guidelines (*p* = 0.13) (see Table S6). We calculated an effect size for 67 of the 80 studies included in this review (see Table S7). In the multivariable regression analysis, the total reporting score did not change after adjusting for the timing of the publication (pre-/post-guideline), the impact factor of the journal, reported effect size, evaluation type, or study design. Of note, we did not find a statistical correlation between the total reporting score and reported effect size (beta coefficient: −2.86; 95% CI: −5.77 to 0.04) (Table 2 and Table 3). Some of the articles published in the 3–6 months after October 2010 (when the CBPR guidelines were posted) might have already been under review by the time the guidelines were published, and therefore could not have benefited from the published guidelines at the time of writing their research article. Thus, we conducted a sensitivity analysis to reclassify the articles that were published in the six months after October 2010 into the pre-guideline group. This resulted in re-classification of two studies. However, our sensitivity analysis did not affect our conclusions.

## 4. Discussion

In this study, we assessed the current reporting quality of articles on CBPR for smoking cessation and evaluated the association between the reporting quality and the publication date in relation to the publication of the reporting guideline. The overall reporting quality was low, and the reporting quality did not improve after the publication of the reporting guideline. The reporting quality was not associated with the reported effect size.

We found an improvement in the reporting quality of only one item out of the 13 items (7.7%). By way of contrast, an evaluation of the reporting quality of RCTs before and after publication of the CONSORT Statement found an improvement in 25 of the 27 items (92.6%) [8], and another evaluation of the quality of reporting before and after the publication of the PRISMA Statement found an improvement in 73.0% of the items [3]. The quality of reporting of the systematic reviews and meta-analyses has not improved as much as the quality of reporting the results of RCTs, probably because the PRISMA guidelines were published more recently than the CONSORT guidelines (2009 vs. 1996).

We believe that there are also a number of reasons for the low reporting quality of CBPR studies found in our study including the lack of awareness by researchers of the guidelines (for example, on Google Scholar, the guideline has so far garnered a total of 70 citations) as well as lack of awareness and use of the guidelines by reviewers and editors during peer review.

In this study, we identified aspects of the reporting quality of articles on CBPR for smoking cessation that could potentially be improved. Some items on the checklist had low reporting frequencies, even after the publication of the reporting guideline. It is crucial that the reporting of participation (q2.3 and q2.4 in Table 1) be improved because the meaning of participation varies in CBPR [11]. Regarding other items with low reporting frequencies, reporting information about the researchers (q3.1 and q3.2) would inform readers of the researchers’ backgrounds. A guideline for reporting the results of qualitative studies specifies that researchers’ characteristics (including personal attribution, relationship with participants) should be reported because they influence the results [20]. Regarding personal outcomes (q3.3), some articles only reported that researchers held informal small group discussions [21,22]. If provided with this information, readers would gain a better understanding of the project including the CBPR process. Regarding the management against challenges, pitfalls, and limitations (q4.2), such information would be useful for improving the study design and smoothing the conduct of future studies.

In contrast to the CONSORT and PRISMA Statements, the CBPR reporting guideline is yet to be broadly endorsed by journals. The lack of improvement in the reporting quality of the results of CBPR following the publication of the CBPR guideline may be because the guideline has not been endorsed by journal editors. Therefore, we recommend that journal editors endorse the CBPR reporting guideline.

### Limitations

There are some limitations to this study. First, the availability of other reporting guidelines could be a confounder in the assessment of a possible association between the publication date of the studies included in this review and the publication of the CBPR reporting guideline, because the other reporting guidelines may have influenced the way that the results of CBPR were reported. For example, the STROBE Statement [23], published in 2007, recommends using graphics. The higher proportion of articles with graphics of the project design (q2.8) may be due to the publication of other reporting guidelines, and not the CBPR reporting guideline. Additionally, regarding the high proportion of articles with graphics on the project design in the period after publication of the reporting guideline, there is a risk of observer bias [24]. Second, in this study, we included studies with different study designs in the analysis and did not perform any subgroup analyses according to the study design. Before-and-after studies can overestimate the effect of an intervention due to the Pygmalion effect [25]. As a result, our calculation method may have overestimated the effect of the reporting guideline. Third, the author of the reporting guideline had better have included the Delphi method process in the process of its establishment for the improvement of its quality. Only 25% (62/244) of the reporting guidelines on the Enhancing the QUALITY and Transparency of health Research (EQUATOR) Network have adopted this process [26]. Fourth, the reviewers were not blinded to the publication date. An item about the timeframe of the study is one of the checklist items (q2.2) in the guidelines, therefore blinding with respect to the timing of publication was deemed to not be practical.

In addition, we only included articles written in English. This review study aimed to assess the impact of the CBPR reporting guideline, and this guideline has not been translated into any languages other than English. Due to this, the publication of the CBPR reporting guideline is less likely to have an impact on articles written in languages other than English, and so including articles written in other languages would not have contributed to assessing the impact of the reporting guideline.

Furthermore, this study focused on CBPR in the area of smoking cessation. We speculate that a similar result would be found if reviews were conducted on the reporting quality of CBPR on other topics. The overall reporting quality of CBPR studies vary, which was the motivation for developing a guideline [11]. It would be useful to assess the reporting quality of CBPR for conditions such as cancer and lifestyle-related diseases to determine whether reporting quality is also low for CBPR on topics other than smoking cessation.

Despite the above limitations, our study has its strengths. To our knowledge, this study is the first systematic review on the impact of the CBPR reporting guideline on the quality of reporting the results of CBPR interventions for smoking cessation.

## 5. Conclusions

Although a CBPR reporting guideline has been published, it has not had an impact on the quality of reporting CBPR on smoking cessation. We recommend that journal editors should endorse the CBPR reporting guideline to encourage its use by more researchers.

## Figures and Tables

**Table 1:** Frequency of use of each reporting item.

	All Studies (*N* = 80)		Studies Pre-Guideline (*n* = 38)		Studies Post-Guideline (*n* = 42)	
Item	*n*	%	*N*	%	*n*	%
1. Structure of the article						
q1: Organizational structure	80	100%	38	100%	42	100%
2. Key elements of the project						
q2.1: Initiation	62	78%	27	71%	35	83%
q2.2: Timeframe	75	94%	36	95%	39	93%
q2.3: Characteristics of participants and co-researchers	59	74%	28	74%	31	74%
q2.4: Role of participants and co-researchers	57	71%	26	68%	31	74%
q2.5: Process/methodology of the project	80	100%	38	100%	42	100%
q2.6: Outcomes/emergent actions	80	100%	38	100%	42	100%
q2.7: Future orientation	72	90%	33	87%	39	93%
q2.8: Graphics of the project design	30	38%	8	21%	22	52%
3. Role of the researchers described						
q3.1: Who wrote the article	17	21%	6	16%	11	26%
q3.2: Who did not write the article	31	39%	17	45%	14	33%
q3.3: Personal outcomes	18	23%	9	24%	9	21%
4. Challenges, pitfalls, and limitations of the project						
q4.1: Their characteristics	74	93%	33	87%	41	98%
q4.2: Their management	38	48%	17	45%	21	50%

**Table 2:** Characteristics of the studies calculated the effect size.

	Studies Pre-Guideline (*n* = 29)		Studies Post-Guideline (*n* = 38)	
Characteristics	Mean	95% CI *	Mean	95% CI *
Reporting score	9.97	1.02, 1.98	9.32	1.39, 2.67
Effect size	0.11	0.12, 0.26	0.08	0.08, 0.16
**Characteristics**	n	%	n	%
Evaluation of the project				
No	13	45%	17	45%
Study design				
Individual randomized CT	4	14%	11	29%
Cluster-randomized CT	10	34%	5	13%
Quasi-experimental	15	52%	22	58%

Abbreviations: CI, confidential interval; CT, controlled trial; * 95% CI for the multiple regression analysis.

**Table 3:** Relationship between the study characteristics and the total reporting score.

Characteristics	Beta Coefficient	95% CI *
Period		
Pre-guideline	ref	—
Post-guideline	−0.83	−1.75, 0.10
Effect size	−2.90	−5.77, 0.04
Evaluation of the project		
No	ref	—
Yes	−0.04	−0.95, 0.88
Study design		
Individually randomized CT	ref	—
Cluster-randomized CT	−0.61	−2.01, 0.79
Quasi-experimental	−0.89	−2.01, 0.23

Abbreviations: CI, confidential interval; CT, controlled trial; ref, reference group; * 95% CI for the multiple regression analysis.

## Supplementary Materials

The following are available online at https://www.mdpi.com/1660-4601/17/11/3898/s1, Appendix S1: Search strategy, Appendix S2: Community-based participatory research reporting guideline checklist, Appendix S3: Eligibility criteria for the items in the checklist, Table S4: List of reviewed studies, Table S5: Characteristics of the studies reviewed, Table S6: The total reporting score according to the article publication period, Table S7: The description of the studies reviewed.
