# Supplementary material for: Reporting Guidelines for Community-Based Participatory Research Did Not Improve the Reporting Quality of Published Studies: A Systematic Review of Studies on Smoking Cessation
Source: Int J Environ Res Public Health. 2020 May 31;17(11):3898. doi: 10.3390/ijerph17113898 (PMC7312250; doi:10.3390/ijerph17113898)
Supplement: Supplementary file 1 [file ijerph-17-03898-s001.zip › S1_Appendix_fin.docx]

**S1 Appendix. Search strategy.**

**MEDLINE**

#1 Community-Based Participatory Research[mh]

#2 community based participatory[tiab]

#3 cbpr[tiab]

#4 action research[tiab]

#5 participatory action[tiab]

#6 participatory research[tiab]

#7 participatory approach*[tiab]

#8 participatory intervention[tiab]

#9 participatory evaluation[tiab]

#10 participatory project[tiab]

#11 participatory program*[tiab]

#12 participatory learning and action[tiab]

#13 Community Based Research[tiab]

#14 Community based approach*[tiab]

#15 community based partnership[tiab]

#16 #1 OR #2 OR #3 OR #4 OR #5 OR #6 OR #7 OR #8 OR #9 OR #10 OR #11 OR #12 OR #13 OR #14 OR #15

#17 smoking cessation[mh]

#18 Tobacco Use Cessation[mh]

#19 smoking[tiab]

#20 tobacco[tiab]

#21 cigar*[tiab]

#22 cigarette*[tiab]

#23 #17 OR #18 OR #19 OR #20 OR #21 OR#22

#24 #16 AND #23

**EMBASE**

S1 EMB.EXACT.EXPLODE("participatory research")

S2 ab(community based participatory) OR ti(community based participatory)

S3 ab(cbpr) OR ti(cbpr)

S4 ab(action research) OR ti(action research)

S5 ab(participatory action) OR ti(participatory action)

S6 ab(participatory research) OR ti(participatory research)

S7 ab(participatory approach*) OR ti(participatory approach*)

S8 ab(participatory intervention) OR ti(participatory intervention)

S9 ab(participatory evaluation) OR ti(participatory evaluation)

S10 ab(participatory project) OR ti(participatory project)

S11 ab(participatory program*) OR ti(participatory program*)

S12 ab(participatory learning and action) OR ti(participatory learning and action)

S13 ab(Community Based Research) OR ti(Community Based Research)

S14 ab(Community based approach*) OR ti(Community based approach*)

S15 ab(community based partnership) OR ti(community based partnership)

S16 S15 OR S14 OR S13 OR S12 OR S11 OR S10 OR S9 OR S8 OR S7 OR S6 OR S5 OR S4 OR S3 OR S2 OR S1

S17 EMB.EXACT.EXPLODE("smoking cessation")

S18 ab(smoking) OR ti(smoking)

S19 ab(tobacco) OR ti(tobacco)

S20 ab(cigar*) OR ti(cigar*)

S21 ab(cigarette*) OR ti(cigarette*)

S22 S21 OR S20 OR S19 OR S18 OR S17

S23 S14 AND S25

remove duplicates

Exclude: Document type: Conference Abstract; Review; Editorial; Conference Review

**CINAHL**

S1 (MH "Action Research")

S2 TI community based participatory OR AB community based participatory

S3 TI cbpr OR AB cbpr

S4 TI action research OR AB action research

S5 TI participatory action OR AB participatory action

S6 TI participatory research OR AB participatory research

S7 TI participatory approach* OR AB participatory approach*

S8 TI participatory intervention OR AB participatory intervention

S9 TI participatory evaluation OR AB participatory evaluation

S10 TI participatory project OR AB participatory project

S11 TI participatory program* OR AB participatory program*

S12 TI participatory learning and action OR AB participatory learning and action

S13 TI Community Based Research OR AB Community Based Research

S14 TI Community based approach* OR AB Community based approach*

S15 TI community-based partnership OR AB community-based partnership

S16 S1 OR S2 OR S3 OR S4 OR S5 OR S6 OR S7 OR S8 OR S9 OR S10 OR S11 OR S12 OR S13 OR S14 OR S15

S17 MH "Smoking Cessation"

S18 MH "Smoking Cessation Programs"

S19 TI smoking OR AB smoking

S20 TI tobacco OR AB tobacco

S21 TI cigar* OR AB cigar*

S22 TI cigarette* OR AB cigarette*

S23 S17 OR S18 OR S19 OR S20 OR S21 OR S22

S24 S16 AND S23
