# Supplementary material for: Reporting Guidelines for Community-Based Participatory Research Did Not Improve the Reporting Quality of Published Studies: A Systematic Review of Studies on Smoking Cessation
Source: Int J Environ Res Public Health. 2020 May 31;17(11):3898. doi: 10.3390/ijerph17113898 (PMC7312250; doi:10.3390/ijerph17113898)
Supplement: Supplementary file 1 [file ijerph-17-03898-s001.zip › S2_Appendix_fin.docx]

**S2 Appendix.** **Community-based participatory research reporting guideline checklist.**

| Item | Content |
| --- | --- |
|  | **Plan ahead for organizational structure** |
| q1 | Adapt conventional organizational headings, or  Consider deriving organizational structure from project design elements or emergent themes, or  Consider a chronological or narrative framework |
|  | **Convey the key elements of the project** |
| q2.1 | How was the project initiated? |
| q2.2 | What was the project’s timeframe? |
| q2.3 | Who were the participants and/or co-researchers? |
| q2.4 | What was the extent of their participation and the nature of their roles? |
| q2.5 | What was the process within and/or the methodology of the project? |
| q2.6 | What were the project outcomes and/or emergent actions? |
| q2.7 | What comes next (if the project is ongoing)? |
| Q2.8 | Consider charts, timelines, tables, or other graphics to convey part or all of the project design |
|  | **Convey the experiences of co-researchers** |
| q3.1 | Pay attention to who is writing the article and how their voices and experiences are represented |
| q3.2 | Pay attention to who is not writing the article and how their voices and experiences are represented |
| q3.3 | What were the personal outcomes of the project? |
|  | **Address the challenges, pitfalls, and limitations of the project^*^** |
| q4.1 | What were they? |
| q4.2 | How were they managed? |

^*^ We did not use the “What can we learn?” question in this review.
