# Supplementary material for: Reporting Guidelines for Community-Based Participatory Research Did Not Improve the Reporting Quality of Published Studies: A Systematic Review of Studies on Smoking Cessation
Source: Int J Environ Res Public Health. 2020 May 31;17(11):3898. doi: 10.3390/ijerph17113898 (PMC7312250; doi:10.3390/ijerph17113898)
Supplement: Supplementary file 1 [file ijerph-17-03898-s001.zip › S3_Appendix_fin.docx]

**S3 Appendix. List of the articles reviewed**

1. Al Hamarneh YN, Tsuyuki RT, Jones CA, Manns B, Tonelli M, Scott-Douglass N, et al. Effectiveness of pharmacist interventions on cardiovascular risk in patients with CKD: A subgroup analysis of the randomized controlled RxEACH trial. Am J Kidney Dis. 2018; 71(1):42–51. doi.org/10.1053/j.ajkd.2017.07.012

2. Allen ML, Hurtado GA, Garcia-Huidobro D, Davey C, Forster J, Reynoso U, et al. Cultural contributors to smoking susceptibility outcomes among Latino youth: The Padres Informados/Jovenes Preparados participatory trial. Fam Community Heal. 2017; 40(2):170–179. https://doi.org/10.1097/FCH.0000000000000147

3. Andrews JO, Mueller M, Dooley M, Newman SD, Magwood GS, Tingen MS. Effect of a smoking cessation intervention for women in subsidized neighborhoods: A randomized controlled trial. Prev Med (Baltim). 2016; 90:170–176. https://doi.org/10.1016/j.ypmed.2016.07.008

4. Anthony D, Dyson PA, Lv J, Thankappan KR, Champgane B, Matthews DR. Community Interventions for Health can support clinicians in advising patients to reduce tobacco use, improve dietary intake and increase physical activity. J Clin Nurs. 2016; 25(21–22):3167–3175. https://doi.org/10.1111/jocn.13323

5. Baghaei A, Sarrafzadegan N, Rabiei K, Gharipour M, Tavasoli AA, Shirani S, et al. How effective are strategies for non-communicable disease prevention and control in a high risk population in a developing country? Isfahan Healthy Heart Programme. Arch Med Sci. 2010; 6(1):24–31. https://doi.org/10.5114/aoms.2010.13503

6. Bauld L, Chesterman J, Ferguson J, Judge K. A comparison of the effectiveness of group-based and pharmacy-led smoking cessation treatment in Glasgow. Addiction. 2009; 104(2):308–316. https://doi.org/10.1111/j.1360-0443.2008.02446.x

7. Berman BA, Gritz ER, Braxton-Owens H, Nisenbaum R. Targeting adult smokers through a multi-ethnic public school system. J Cancer Educ. 1995; 10(2):91–101. PMID: 7669541

8. Biglan A, Ary DV, Smolkowski K, Duncan T, Black C. A randomised controlled trial of a community intervention to prevent adolescent tobacco use. Tob Control. 2000; 9(1):24–32. https://doi.org/10.1136/tc.9.1.24

9. Bryce A, Butler C, Gnich W, Sheehy C, Tappin DM. CATCH: development of a home-based midwifery intervention to support young pregnant smokers to quit. Midwifery. 2009; 25(5):473–482. https://doi.org/10.1016/j.midw.2007.10.006

10. Carlson LE, Taenzer P, Koopmans J, Bultz BD. Eight-year follow-up of a community-based large group behavioral smoking cessation intervention. Addict Behav. 2000; 25(5): 725–741. https://doi.org/10.1016/s0306-4603(00)00081-2

11. Carlson LE, Taenzer P, Koopmans J, Casebeer A. Predictive value of aspects of the Transtheoretical Model on smoking cessation in a community-based, large-group cognitive behavioral program. Addict Behav. 2003; 28(4): 725–740. https://doi.org/10.1016/s0306-4603(01)00268-4

12. Colby SM, Nargiso J, Tevyaw TO, Barnett NP, Metrik J, Lewander W, et al. Enhanced motivational interviewing versus brief advice for adolescent smoking cessation: Results from a randomized clinical trial. Addict Behav. 2012; 37(7):817–823. https://doi.org/10.1016/j.addbeh.2012.03.011

13. Connolly SB, Kotseva K, Jennings C, Atrey A, Jones J, Brown A, et al. Outcomes of an integrated community-based nurse-led cardiovascular disease prevention programme. Heart. 2017; 103(11):840–847. https://doi.org/10.1136/heartjnl-2016-310477

14. Cornell CE, Littleton MA, Greene PG, Pulley L, Brownstein JN, Sanderson BK, et al. A community health advisor program to reduce cardiovascular risk among rural African-American women. Health Educ Res. 2009; 24(4):622–633. https://doi.org/10.1093/her/cyn063

15. Cutrona SL, Sadasivam RS, DeLaughter K, Kamberi A, Volkman JE, Cobb N, et al. Online tobacco websites and online communities—who uses them and do users quit smoking? The quit-primo and national dental practice-based research network Hi-Quit studies. Transl Behav Med. 2016; 6(4):546–557. https://doi.org/10.1007/s13142-015-0373-5

16. Doyle D, Tommarello C, Broce M, Emmett M, Pollard C. Implementation and outcomes of a community-based pulmonary rehabilitation program in rural Appalachia. J Cardiopulm Rehabil Prev. 2017; 37(4):295–298. https://doi.org/10.1097/hcr.0000000000000247

17. Elder JP, McGraw SA, Rodrigues A, Lasater TM, Ferreira A, Kendall L, et al. Evaluation of two community-wide smoking cessation contests. Prev Med (Baltim). 1987; 16(2):221–234. https://doi.org/10.1016/0091-7435(87)90086-7

18. Elsey H, Khanal S, Manandhar S, Sah D, Baral SC, Siddiqi K, et al. Understanding implementation and feasibility of tobacco cessation in routine primary care in Nepal: A mixed methods study. Implement Sci. 2016; 11:104. https://doi.org/10.1186/s13012-016-0466-7

19. English KC, Merzel C, Moon-Howard J. Translating public health knowledge into practice: development of a lay health advisor perinatal tobacco cessation program. J Public Heal Manag Pract. 2010; 16(3):E9–19. https://doi.org/10.1097/phh.0b013e3181af6387

20. Flewelling RL, Austin D, Hale K, LoPlante M, Liebig M, Piasecki L, et al. Implementing research-based substance abuse prevention in communities: Effects of a coalition-based prevention initiative in Vermont. J Community Psychol. 2005; 33(3):333–353. https://doi.org/10.1002/jcop.20052

21. Froelicher ES, Doolan D, Yerger VB, McGruder CO, Malone RE. Combining community participatory research with a randomized clinical trial: The Protecting the Hood Against Tobacco (PHAT) smoking cessation study. Hear Lung J Acute Crit Care. 2010; 39(1):50–63.

22. Gondnale G, Ingole A, Gaidhane A. Effectiveness of text messages for positive change in behaviour amongst young adult tobacco users in rural Wardha: Quasi-experimental study. Indian J Public Heal Res Dev. 2017; 8(4):657–661. https://doi.org/10.5958/0976-5506.2017.00412.0

23. Graham AL, Papandonatos GD, Cha S, Erar B, Amato MS, Cobb NK, et al. Improving adherence to smoking cessation treatment: Intervention effects in a web-based randomized trial. Nicotine Tob Res. 2017; 19(3):324–332. https://doi.org/10.1093/ntr/ntw282

24. Groth-Marnat G, Leslie S, Renneker M. Tobacco control in a traditional Fijian village: Indigenous methods of smoking cessation and relapse prevention. Soc Sci Med. 1996; 43(4):473–7.47. https://doi.org/10.1016/0277-9536(95)00425-4

25. Herbert RJ, Gagnon AJ, O’Loughlin JL, Rennick JE. Testing an empowerment intervention to help parents make homes smoke-free: A randomized controlled trial. J Community Health. 2011; 36(4):650–657. https://doi.org/10.1007/s10900-011-9356-8

26. Jayakrishnan R, Uutela A, Mathew A, Auvinen A, Mathew PS, Sebastian P. Smoking cessation intervention in rural Kerala, India: Findings of a randomised controlled trial. Asian Pacific J Cancer Prev. 2013; 14(11):6797–6802. https://doi.org/10.7314/apjcp.2013.14.11.6797

27. Khan N, Anderson JR, Du J, Tinker D, Bachyrycz AM, Namdar Dr. R. Smoking cessation and its predictors: Results from a community-based pharmacy tobacco cessation program in New Mexico. Ann Pharmacother. 2012; 46(9):1198–1204. https://doi.org/10.1345/aph.1p146

28. Kim S, Koniak-Griffin D, Flaskerud JH, Guarnero PA. The impact of lay health advisors in cardiovascular health promotion: Using a community-based participatory approach. J Cardiovasc Nurs. 2004; 19(3):192–199. https://doi.org/10.1097/00005082-200405000-00008

29. Maguire TA, McElnay JC, Drummond A. A randomized controlled trial of a smoking cessation intervention based in community pharmacies. Addiction. 2001; 96(2):325–331. https://doi.org/10.1046/j.1360-0443.2001.96232516.x

30. Marin BV, Perez-Stable EJ, Marin G, Hauck WW. Effects of a community intervention to change smoking behavior among Hispanics. Am J Prev Med. 1990; 10(6):340–347. PMID: 7880553

31. Matone M, O’Reilly AL, Luan X, Localio R, Rubin DM. Home visitation program effectiveness and the influence of community behavioral norms: a propensity score matched analysis of prenatal smoking cessation. BMC Public Health. 2012; 12:1016. https://doi.org/10.1186/1471-2458-12-1016

32. Matthews AK, Li C-C, Kuhns LM, Tasker TB, Cesario JA. Results from a community-based smoking cessation treatment program for LGBT smokers. J Environ Public Health. 2013; 2013:984508. https://doi.org/10.1155/2013/984508

33. McDermott RA, Schmidt B, Preece C, Owens V, Taylor S, Li M, et al. Community health workers improve diabetes care in remote Australian indigenous communities: Results of a pragmatic cluster randomized controlled trial. BMC Health Serv Res. 2015; 15:68. https://doi.org/10.1186/s12913-015-0695-5

34. McDonnell DD, Kazinets G, Lee HJ, Moskowitz JM. An internet-based smoking cessation program for Korean Americans: Results from a randomized controlled trial. Nicotine Tob Res. 2011; 13(5):336–343. https://doi.org/10.1093/ntr/ntq260

35. Mendenhall T, Harper P, Stephenson H, Haas GS. The SANTA project (Students Against Nicotine and Tobacco Addiction): Using community-based participatory research to reduce smoking in a high-risk young adult population. Action Res. 2011; 9(2):199–213. https://doi.org/10.1177/1476750310388051

36. Moore L, Campbell R, Whelan A, Mills N, Lupton P, Misselbrook E, et al. Self help smoking cessation in pregnancy: cluster randomised controlled trial. BMJ. 2002; 325(7377):1383. https://doi.org/10.1136/bmj.325.7377.1383

37. Moskowitz JM, McDonnell DD, Kazinets G, Lee HJ. Online smoking cessation program for Korean Americans: Randomized trial to test effects of incentives for program completion and interim surveys. Prev Med (Baltim). 2016; 86:70–76. https://doi.org/10.1016/j.ypmed.2016.01.019

38. Muhajarine N, Ng J, Bowen A, Cushon J, Johnson S. Understanding the impact of the Canada Prenatal Nutrition Program: A quantitative evaluation. Can J Public Heal. 2012; 103:S26–31. https://doi.org/10.1007/bf03404456

39. Nafziger AN, Erb TA, Jenkins PL, Lewis C, Pearson TA. The Otsego-Schoharie Healthy Heart program: Prevention of cardiovascular disease in the rural US. Scand J Public Health Suppl. 2001; 56:21–32. https://doi.org/10.1177/14034948010290021501

40. Nguyen QN, Pham ST, Nguyen VL, Weinehall L, Wall S, Bonita R, et al. Effectiveness of community-based comprehensive healthy lifestyle promotion on cardiovascular disease risk factors in a rural Vietnamese population: A quasi-experimental study. BMC Cardiovasc Disord. 2012; 12(1):56. https://doi.org/10.1186/1471-2261-12-56

41. Nierkens V, Kunst AE, Vries H De, Voorham TAJ, Stronks K. Reach and effectiveness of a community program to reduce smoking among ethnic Turkish residents in Rotterdam, the Netherlands: A quasi-experimental design. Nicotine Tob Res. 2013; 15(1):112–120. https://doi.org/10.1093/ntr/nts096

42. Nilsson M, Stenlund H, Bergström E, Weinehall L, Janlert U. It takes two: Reducing adolescent smoking uptake through sustainable adolescent-adult partnership. J Adolesc Heal. 2006; 39(6):880–886. https://doi.org/10.1016/j.jadohealth.2006.07.004

43. O’Riordan DL, Nicole S, Pedro H-A. A community-based approach to tobacco prevention: Hawaii’s youth taking on the tobacco industry. Hawaii Med J. 2007; 64:310–312. PMID: 16379225

44. Olaiya MT, Cadilhac DA, Kim J, Nelson MR, Srikanth VK, Gerraty RP, et al. Community-based intervention to improve cardiometabolic targets in patients with stroke: A randomized controlled trial. Stroke. 2017; 48(9):2504–2510. https://doi.org/10.1161/strokeaha.117.017499

45. Pansu P, Lima L, Fointiat V. When saying no leads to compliance: The door-in-the-face technique for changing attitudes and behaviors towards smoking at work. Rev Eur Psychol Appl. 2014; 64(1):19–27. https://doi.org/10.1016/j.erap.2013.11.001

46. Pentz MA, Flay BR, Daniels S, Cormack C, Dwyer JH, Johnson CA. Effects of program implementation on adolescent drug use behavior: The Midwestern Prevention Project (MPP). Eval Rev. 1990; 14(3):264–289. https://doi.org/10.1177/0193841x9001400303

47. Perry CL, Klepp K-I, Sillers C. Community-wide strategies for cardiovascular health: The Minnesota Heart Health Program youth program. Health Educ Res. 1989; 4(1):87–101. https://doi.org/10.1093/her/4.1.87

48. Perry CL, Kelder SH, Murray DM, Klepp K-I. Communitywide smoking prevention: Long-term outcomes of the Minnesota Heart Health Program and the Class of 1989 Study. Am J Public Health. 1992; 82(9):1210–1216. https://doi.org/10.2105/ajph.82.9.1210

49. Rabius V, Wiatrek D, McAlister AL. African American participation and success in telephone counseling for smoking cessation. Nicotine Tob Res. 2012; 14(2):240–242. https://doi.org/10.1093/ntr/ntr129

50. Rivas C, Sohanpal R, Macneill V, Steed L, Edwards E, Antao L, et al. Determining counselling communication strategies associated with successful quits in the National Health Service community pharmacy Stop Smoking programme in East London: A focused ethnography using recorded consultations. BMJ Open. 2017; 7(10):e015664. https://doi.org/10.1136/bmjopen-2016-015664

51. Schinke SP, Tepavac L, Cole KC. Preventing substance use among Native American youth: Three-year results. Addict Behav. 2000; 25(3):387–397. https://doi.org/10.1016/s0306-4603(99)00071-4

52. Schoenberg NE, Studts CR, Shelton BJ, Liu M, Clayton R, Bispo JB, et al. A randomized controlled trial of a faith-placed, lay health advisor delivered smoking cessation intervention for rural residents. Prev Med Rep. 2016;3:317–323. https://doi.org/10.1016/j.pmedr.2016.03.006

53. Share M, Quinn M, Ryan C. Evaluation of a 5-year school-based county-wide smoking education programme. Ir Med J. 2004; 97(9):264–247. PMID: 15568582.

54. Utz SW, Shuster GF, Merwin E, Williams B. A community-based smoking-cessation program: Self-care behaviors and success. Public Health Nurs. 1994;11(5): 291–299. https://doi.org/10.1111/j.1525-1446.1994.tb00190.x

55. Sheikhattari P, Apata J, Kamangar F, Schutzman C, O’Keefe A, Buccheri J, et al. Examining smoking cessation in a community-based versus clinic-based intervention using community-based participatory research. J Community Health. 2016; 41(6):1146–1152. https://doi.org/10.1007/s10900-016-0264-9

56. Shlay JC, Barber B, Mickiewicz T, Maravi M, Drisko J, Estacio R, et al. Reducing cardiovascular disease risk using patient navigators, Denver, Colorado, 2007–2009. Prev Chronic Dis. 2011; 8(6):A143. PMID: 22005636

57. Shuster GF, Utz SW, Merwin E. Implementation and outcomes of a community-based self-help smoking cessation program. J Community Health Nurs. 1996; 13(3):187–198. https://doi.org/10.1207/s15327655jchn1303_6

58. Slater MD, Kelly KJ, Edwards RW, Thurman PJ, Plested BA, Keefe TJ, et al. Combining in-school and community-based media efforts: Reducing marijuana and alcohol uptake among younger adolescents. Health Educ Res. 2006; 21(1):157–167. https://doi.org/10.1093/her/cyh056

59. Snaterse M, Jorstad HT, Minneboo M, Lachman S, Boekholdt SM, ter Riet G, et al. Smoking cessation after nurse-coordinated referral to a comprehensive lifestyle programme in patients with coronary artery disease: a substudy of the RESPONSE-2 trial. Eur J Cardiovasc Nurs. 2019; 18(2):113–121. https://doi.org/10.1177/1474515118795722

60. Stein-Seroussi A, Stockton L, Brodish P, Meyer M. Randomized controlled trial of the ACTION smoking cessation curriculum in tobacco-growing communities. Addict Behav. 2009; 34(9):737–743. https://doi.org/10.1016/j.addbeh.2009.04.016

61. Thompson KA, Parahoo AK, Blair N. A nurse-led smoking cessation clinic — Quit rate results and views of participants. Health Educ J. 2007; 66(4):307–322. https://doi.org/10.1177/0017896907083151

62. Vaid I, Ahmed K, May D, Manheim D. The WISEWOMAN program: Smoking prevalence and key approaches to smoking cessation among participants, July 2008–June 2013. J Women’s Heal. 2014; 23(4):288–295. https://doi.org/10.1089/jwh.2013.4712

63. Vartiainen E, Pennanen M, Haukkala A, Dijk F, Lehtovuori R, De Vries H. The effects of a three-year smoking prevention programme in secondary schools in Helsinki. Eur J Public Health. 2007; 17(3):249–256. https://doi.org/10.1093/eurpub/ckl107

64. Velasquez MM, von Sternberg KL, Floyd RL, Parrish D, Kowalchuk A, Stephens NS, et al. Preventing alcohol and tobacco exposed pregnancies: CHOICES Plus in Primary Care. Am J Prev Med. 2017; 53(1):85–95. https://doi.org/10.1016/j.amepre.2017.02.012

65. Vial RJ, Jones TE, Ruffin RE, Gilbert AL. Smoking cessation program using nicotine patches linking hospital to the community. J Pharm Pract Res. 2002; 32(1):57–62. https://doi.org/10.1002/jppr200232157

66. Darity WA, Chen TTL, Tuthill RW, Buchanan DR, Winder AE, Stanek E, et al. A multi-city community based smoking research intervention project in the African-American population. Int Q Community Health Educ. 1997; 17(2):117–130. https://doi.org/10.2190/cexy-wg7c-gl3e-a2bp

67. Wadland WC, Stöffelmayr B, Berger E, Crombach A, Ives K. Enhancing smoking cessation rates in primary care. J Fam Pract. 1999; 48(9):711–718. PMID: 10498078

68. Wagner FA, Sheikhattari P, Buccheri J, Gunning M, Bleich L, Schutzman C. A community-based participatory research on smoking cessation intervention for urban communities. J Health Care Poor Underserved. 2016; 27(1):35–50. https://doi.org/10.1353/hpu.2016.0017

69. Wewers ME, Shoben A, Conroy S, Curry E, Ferketich AK, Murray DM, et al. Effectiveness of two community health worker models of tobacco dependence treatment among community residents of Ohio Appalachia. Nicotine Tob Res. 2017; 19(12):1499–1507. https://doi.org/10.1093/ntr/ntw265

70. Woodruff SI, Conway TL, Edwards CC, Elliott SP, Crittenden J. Evaluation of an Internet virtual world chat room for adolescent smoking cessation. Addict Behav. 2007; 32(9):1769–1786. https://doi.org/10.1016/j.addbeh.2006.12.008

71. Wu Y, Stanton BF, Galbraith J, Kaljee L, Cottrell L, Li X, et al. Sustaining and broadening intervention impact: A longitudinal randomized trial of 3 adolescent risk reduction approaches. Pediatrics. 2003; 111(1):e32–e38. https://doi.org/10.1542/peds.111.1.e32

72. Xiangyang T, Lan Z, Xueping M, Tao Z, Yuzhen S, Jagusztyn M. Beijing health-promoting universities: Practice and evaluation. Health Promot Int. 2003; 18(2):107–113. https://doi.org/10.1093/heapro/18.2.107

73. Apata J, Sheikhattari P, Bleich L, Kamangar F, O'Keefe AM, Wagner FA. Addressing tobacco use in underserved communities through a peer-facilitated smoking cessation program. Journal of community health 44.5 (2019): 921-931. https://doi.org/10.1007/s10900-019-00635-8

74. Joshi R, Agrawal T, Fathima F, Usha T, Thomas T, Misquith D, et al. Cardiovascular risk factor reduction by community health workers in rural India: A cluster randomized trial. Am Heart J. 216 (2019):9–19. https://doi.org/10.1016/j.ahj.2019.06.007

75. Khetan A, Zullo M, Rani A, Gupta R, Purushothaman R, Bajaj NS, et al. Effect of a community health worker-based approach to integrated cardiovascular risk factor control in India: A cluster-randomized controlled trial. Glob Heart. 2019; 14(4):355–365. https://doi.org/10.1016/j.gheart.2019.08.003

76. Jiang N, Siman N, Cleland CM, Van Devanter N, Nguyen T, Nguyen N, et al. Effectiveness of village health worker-delivered smoking cessation counseling in Vietnam. Nicotine Tob Res. 2019; 21(11):1524-1530. https://doi.org/10.1093/ntr/nty216

77. Snaterse M, Jorstad HT, Minneboo M, Lachman S, Boekholdt SM, ter Riet G, et al. Smoking cessation after nurse-coordinated referral to a comprehensive lifestyle programme in patients with coronary artery disease: a sub-study of the RESPONSE-2 trial. Eur J Cardiovasc Nurs. 2019; 18(2):113–121. https://doi.org/10.1177/1474515118795722

78. Gilbody S, Peckham E, Bailey D, Arundel C, Heron P, Crosland S, et al. Smoking cessation for people with severe mental illness (SCIMITAR+): a pragmatic randomised controlled trial. Lancet Psychiatry. 2019; 6(5):379–390. https://doi.org/10.1016/S2215-0366(19)30047-1

79. Secades-Villa R, López-Núñez C, Weidberg S, González-Roz A, Alonso-Pérez F. A randomized controlled trial of contingency management for smoking abstinence versus contingency management for shaping cessation: One-year outcome. Exp Clin Psychopharmacol. 2019; 27(6):561–568. https://doi.org/10.1037/pha0000269.

80. Bottorff JL, Oliffe JL, Sarbit G, Huisken A, Caperchione C, Anand A, et al. Evaluating the feasibility of a gender-sensitized smoking cessation program for fathers. Psychol Men Masc. 2019; 20(2):194–207. https://doi.org/10.1037/men0000190
