# Supplementary material for: Reporting Guidelines for Community-Based Participatory Research Did Not Improve the Reporting Quality of Published Studies: A Systematic Review of Studies on Smoking Cessation
Source: Int J Environ Res Public Health. 2020 May 31;17(11):3898. doi: 10.3390/ijerph17113898 (PMC7312250; doi:10.3390/ijerph17113898)
Supplement: Supplementary file 1 [file ijerph-17-03898-s001.zip › S4_Table_fin.docx]

**S4 Table.** **Characteristics of the studies reviewed.**

| **Study characteristics expressed as frequencies and proportions** | | | | | | |  |
| --- | --- | --- | --- | --- | --- | --- | --- |
|  | **Total (N=80)** | | **Pre-guideline (n=38)** | | **Post-guideline (n=42)** | |  |
| **Characteristics** | **n** | **%** | **n** | **%** | **n** | **%** |  |
| Region | | | | | | |  |
| US | 44 | 55 | 25 | 66 | 19 | 45 |  |
| North America (excluding the US) | 6 | 8 | 2 | 5 | 4 | 10 |  |
| Europe | 15 | 19 | 7 | 18 | 8 | 5 |  |
| Asia | 9 | 11 | 2 | 5 | 7 | 17 |  |
| Oceania | 4 | 6 | 2 | 5 | 2 | 6 |  |
| Multiple countries | 1 | 1 | 0 | 0 | 1 | 3 |  |
| World^*^ | 1 | 1 | 0 | 0 | 1 | 3 |  |
| Evaluation of the program^†^ | 44 | 55 | 25 | 66 | 19 | 45 |  |
| Study design |  |  |  |  |  |  |  |
| Individually-randomized CT | 19 | 24 | 7 | 18 | 12 | 29 |  |
| Cluster-randomized CT | 17 | 21 | 8 | 21 | 9 | 21 |  |
| Quasi-experimental study | 44 | 55 | 23 | 61 | 21 | 50 |  |
| **Study characteristics expressed as means and standard deviations** | | | | | | | |
|  | **Total (N=80)** | | **Pre-guideline (n=38)** | | **Post-guideline (n=42)** | | |
|  | Mean | SD | Mean | SD | Mean | SD | |
| Duration^§^ | 3.76 | 3.42 | 4.71 | 4.20 | 2.92 | 2.22 | |
| Number of professionals^**^ | 2.40 | 2.84 | 2.47 | 3.27 | 2.33 | 2.38 | |

SD, standard deviation; CT, controlled trial;

^*^ Internet-based intervention, not limited to a specific country.

^†^ The article reported on an evaluation of a smoking cessation program.

^§^ Duration of the smoking cessation program.

^**^ The number of professionals engaged in providing the smoking cessation intervention.
