# Supplementary material for: Reporting Guidelines for Community-Based Participatory Research Did Not Improve the Reporting Quality of Published Studies: A Systematic Review of Studies on Smoking Cessation
Source: Int J Environ Res Public Health. 2020 May 31;17(11):3898. doi: 10.3390/ijerph17113898 (PMC7312250; doi:10.3390/ijerph17113898)
Supplement: Supplementary file 1 [file ijerph-17-03898-s001.zip › S5_Table_fin.docx]

**S5 Table. The total reporting score according to the article publication period.**

|  | **All studies (N=80)** | | **Published pre-guideline (n=38)** | | **Published post-guideline (n=42)** | |
| --- | --- | --- | --- | --- | --- | --- |
| Total reporting score (mean, SD) | 9.66 | 1.96 | 9.32 | 1.91 | 9.98 | 1.95 |

Abbreviation: SD, standard deviation
