# Supplementary material for: Reporting Guidelines for Community-Based Participatory Research Did Not Improve the Reporting Quality of Published Studies: A Systematic Review of Studies on Smoking Cessation
Source: Int J Environ Res Public Health. 2020 May 31;17(11):3898. doi: 10.3390/ijerph17113898 (PMC7312250; doi:10.3390/ijerph17113898)
Supplement: Supplementary file 1 [file ijerph-17-03898-s001.zip › S6_Table_ver1.1.docx]

**S6 Table. Description of the studies reviewed**

| **No** | **Author & Year** | **Country** | **Study setting** | **Description of the intervention** | **Study design** | **Effect Size** | **Reporting Score** |
| --- | --- | --- | --- | --- | --- | --- | --- |
| 1 | Al Hamarneh et al., 2018 | Canada | Community | Keeping a record of smoking status, making treatment recommendations by pharmacists | IR | 0.196 | 5 |
| 2 | Allen et al., 2017 | US | Rural region receiving many Latino immigrants | Integrating parent and community priorities, family education; eight weekly group-sessions that included role-playing skill-building exercises, and small-group discussions (2.5 hours per session) | CR | N/A, OR: 0.35, 95% CI: 0.16-0.75 | 13 |
| 3 | Andrews et al., 2016 | US | Community | Individual contact with a community health worker; peer-group behavioral sessions; and nicotine replacement for 24 weeks | CR | 0.047 | 10 |
| 4 | Anthony et al., 2016 | China, India, Mexico | Community | Discussions about risk factors for non-communicable disease, assessment of risk factors for non-communicable disease, advice and treatment for smoking cessation | QE/BA | N/A | 10 |
| 5 | Baghaei et al., 2010 | Iran | County | Baseline survey and a targeted community outreach program | QE/CT | 0.133 | 10 |
| 6 | Bauld et al., 2009 | UK | Community | Smoking treatment services provided by pharmacists (one-to-one support), and community-level group support (group-based community support) | IR | 0.169 | 12 |
| 7 | Berman et al., 1995 | US | Secondary schools | Seven 90-minute group sessions on smoking-cessation | CR | −0.009 | 9 |
| 8 | Biglan et al., 2000 | US | High schools | Four-module program consisting of: media advocacy, youth anti-tobacco activities, family communication, and smoking cessation by youth | CR | 0.034 | 11 |
| 9 | Bryce et al., 2009 | UK | Hospital maternity unit | Program with a tailored and holistic approach to smoking cessation, focusing on both individual choices and motivations, an on the wider life circumstances | QE/BA | 0.103 | 14 |
| 10 | Carlson et al., 2000 | Canada | Regional outpatient cancer center | Eight 90-minute group sessions over 4 months with education, self-monitoring, phased nicotine reduction, a group quit date, and behavioral modification techniques | QE/BA | 0.393 | 11 |
| 11 | Carlson et al., 2003 | Canada | Smoking cessation clinic | Eight 90-minute group sessions over 4 months with education, self-monitoring, a group quit date, and behavioral modification techniques | QE/BA | 0.395 | 11 |
| 12 | Colby et al., 2012 | US | High schools, hospitals, clinics | Motivational interviewing by trained counselors, using the principles: rapport, discussion about smoking, feedback, imagination of the smoke-free future, developing a smoking cessation plan, and enhancing the self-efficacy | IR | 0.021 | 9 |
| 13 | Connolly et al., 2017 | UK | Community | Education on smoking cessation | QE/BA | 0.005 | 9 |
| 14 | Cornell et al., 2009 | US | Rural town | Formative evaluation through a community meeting, and a 12-month intervention that included educational events/classes by project staff | QE | N/A | 9 |
| 15 | Cutrona et al., 2016 | world | Web-based community | Online educational content that included family tools (how to get constructive help from family), articles, and health risk information | QE/CT | 0.039 | 9 |
| 16 | Doyle et al., 2017 | US | Rural communities | Twelve-week, 24-session program of graded exercise and education | QE/BA | 0.217 | 10 |
| 17 | Elder et al., 1987 | US | Blue-collar community | Conducting a lottery to develop a risk-reduction program | QE | 0.772 | 9 |
| 18 | Elsey et al., 2016 | Nepal | Two districts (rural/urban) | One-day workshop on behavior change techniques, with workshop participants setting a quit date | QE/BA | 0.370 | 7 |
| 19 | English, et al., 2010 | US | City/metropolitan area | To ask the patient about smoking status, to provide advice to quit with personalized messages about the impact of smoking, to assess the patients' willingness for smoking cessation, to suggest and encourage the use of problem-solving methods and skills for smoking cessation, and to assess smoking status and encourage cessation periodically | QE/BA | 0.109 | 10 |
| 20 | Flewelling et al., 2005 | US | Community | Training program for coalition coordinators to increase efficacy at a community level. The program was endorsed by the Center for Substance Abuse Prevention. | QE | −0.400 | 12 |
| 21 | Froelicher et al., 2010 | US | Low-income community | The researchers prioritized community issues, provided information about tobacco industry tactics, deconstructed media campaigns, and engaged in advocacy (letter writing, leadership groups) | IR | 0.105 | 9 |
| 22 | Gondnale et al., 2017 | India | Villages | Weekly text messages over 6 months providing information about the harmful effects of tobacco | QE/BA | 0.100 | 12 |
| 23 | Graham et al., 2017 | US | Web-based community | Web-based intervention providing advice on problem-solving/coping skills, education about effective smoking-cessation medications and ways to prevent relapse, information on social support, and a series of videos explaining nicotine addiction and medication effects | IR | 0.008 | 11 |
| 24 | Groth-Marnat et al., 1996 | Fiji | Villages | Discussions with community members | QE/BA | N/A | 8 |
| 25 | Herbert et al., 2011 | Canada | Public health nursing offices, family resource centers, and daycare centers | Three 2-hour weekly group sessions with interactive discussion about smoking cessation and participatory experiences, followed by 3weekly telephone calls | IR | −0.076 | 9 |
| 26 | Jayakrishnan et al., 2013 | India | Rural community | Educational materials on the adverse effects of tobacco, and 4 counseling sessions (including a medical camp [group session] and 3 individual counseling sessions with motivational counseling), provided by trained medical social workers | CR | 0.079 | 10 |
| 27 | Khan et al., 2012 | US | Community-based pharmacies | Structured smoking-cessation intervention provided by a pharmacist, which included an initial 30-minute counseling session | QE/BA | 0.250 | 11 |
| 28 | Kim et al., 2004 | US | Underserved Latino community | Three classes on healthy nutrition, physical activity, and maintaining a smoke-free environment | QE/BA | N/A, t = –2.61, p < 0.05 | 11 |
| 29 | Maguire et al., 2001 | UK | Community pharmacies | Structured community pharmacy-based smoking cessation program, consisting of a structured counseling program, an information leaflet, and follow-up | IR | 0.116 | 9 |
| 30 | Marin et al., 1990 | US | Community | Informational and motivational messages provided using a variety of media, including radio and television public service announcements and talk shows, pamphlets, fliers, posters, billboards, external signs on buses, newspaper articles, and bumper stickers | QE/CT | 0.044 | 12 |
| 31 | Matone et al., 2012 | US | Community | Providing a program of prenatal and postpartum home visitation by nurses. | QE/CT | 0.053 | 5 |
| 32 | Matthews et al., 2013 | US | Community | Cost-effective, group-based smoking cessation treatment | QE/CT | -0.122 | 10 |
| 33 | McDermott et al., 2015 | Australia | Remote communities | Structured intervention by community health workers that included discussing the rationale for management/treatment of chronic obstructive pulmonary disease, case management, working in a primary care team, and engaging with families for client self-management | CR | -0.128 | 12 |
| 34 | McDonnell et al., 2011 | US | Online smoking cessation program | Six-domain smoking cessation program based on the cognitive-behavioral theory | IR | -0.016 | 8 |
| 35 | Mendenhall et al., 2011 | US | Secondary school | Developing student teams including non-smokers with professional team members, and providing information and support for smoking cessation | QE/BA | N/A, F:(4,1076) = 0.544, p = 0.70 | 13 |
| 36 | Moore L Whelan A et al., 2002 | UK | NHS hospital trust | Self-help booklets for women in pregnancy, written by a midwife, providing a step-by-step smoking cessation program to increase motivation, and to teach smoking cessation and relapse prevention strategies | IR | -0.019 | 8 |
| 37 | Moskowitz et al., 2016 | US | Internet-based network | Quitting is Winning, a 6-module internet-based, cognitive-behavioral smoking cessation program based on Prochaska's Transtheoretical Model. Short introductory videos were used for each of the modules. | IR | 0.010 | 11 |
| 38 | Muhajarine et al., 2012 | Canada | Community | Providing a smoking cessation program during the pregnancy. | QE | N/A, OR: 1.19, 95% CI: 1.05-1.36 | 7 |
| 39 | Nafziger et al., 2001 | US | Rural county | Community assessment survey and education, referrals, programs, and poster contests | QE | 0.060 | 13 |
| 40 | Nguyen et al., 2012 | Vietnam | Rural communes | Campaigns promoting a healthy lifestyle | QE/CT | 0.006 | 9 |
| 41 | Nierkens et al., 2013 | Netherland | Turkish community | Meeting about smoking cessation, and information about smoking cessation published in a free Turkish newspaper, a poster, and a brochure | QE/BA | 0.017 | 7 |
| 42 | Nilsson et al., 2006 | Sweden | School | Discussion with students and their parents about smoking cessation, and informational meetings | QE/CT | 0.054 | 11 |
| 43 | O’Riordan et al., 2005 | US | Community | Educational programs provided by organizations | QE/BA | 0.131 | 10 |
| 44 | Olaiya et al., 2017 | Australia | Community, tertiary hospitals | Specialist review of care plans and education by nurses for patients of stroke/transient ischemic attack | IR | −0.016 | 9 |
| 45 | Pansu et al., 2014 | France | Hospital staffs | Informing patients of meetings regarding smoking cessation session | QE/CT | 0.085 | 5 |
| 46 | Pentz et al., 1990 | US | The middle or junior high school | Five-component program comprising: 1) a school program, 2) a parent program, 3) community organization, 4) health policy change, and 5) mass media coverage and programming | QE | 0.122 | 10 |
| 47 | Perry et al., 1989 | US | Schools | Providing healthy role models, and social skills training | QE/CT | 0.076 | 9 |
| 48 | Perry et al., 1992 | US | Public high schools | Behavioral health programs based on the Social Influences model, provided through small group discussions | QE/CT | 0.090 | 8 |
| 49 | Rabius et al., 2012 | US | Districts through telephone network focused on African Americans | Telephone interviews to provide counseling for smoking cessation | IR | 0.112 | 8 |
| 50 | Rivas et al., 2017 | UK | Economically disadvantaged community | Consultations to a smoking cessation program and prescribing medications | QE/BA | N/A | 14 |
| 51 | Schinke et al., 2000 | US | Native American communities | Weekly sessions (15–50 minutes) containing instruction, modeling, and rehearsal of cognitive-behavioral skills related to tobacco control | CR | 0.022 | 10 |
| 52 | Schoenberg et al., 2016 | US | Rural counties | Twelve-week smoking cessation program with weekly 90-minute group sessions using the Cooper/Clayton Method Stop Smoking curriculum | CR | 0.251 | 11 |
| 53 | Share et al., 2004 | Ireland | Primary school | Smoke-Free Leitrim Project promoting better smoking behavior, knowledge, and attitudes | IR | 0.130 | 10 |
| 54 | Sharon et al., 1994 | US | Small city and counties | Three-phase program comprising: 1) recruitment of volunteers and involvement of mass media, 2) distribution of materials and 3) implementation of daily radio announcements | QE/BA | 0.151 | 8 |
| 55 | Sheikhattari et al., 2016 | US | Low-income communities | Six-week smoking cessation module (2 weeks for preparation and motivation, and 4 weeks for quitting), followed by a 6-week relapse prevention module. | QE/CT | 0.143 | 11 |
| 56 | Shlay et al., 2011 | US | Community health centers | A one-hour counseling session and follow-up telephone calls | QE/BA | 0.034 | 11 |
| 57 | Shuster III et al., 1996 | US | Small city and county | Three-phase, self-help smoking cessation intervention: 1) Recruitment of volunteers and involving mass media; 2) Distribution of smoking cessation materials; and 3) Radio announcements | QE/BA | 0.151 | 8 |
| 58 | Slater et al., 2006 | US | Middle school | Media campaign, promotional items, workshops | CR | N/A, t = 1.90,(df: 14), p = 0.04 | 10 |
| 59 | Snaterse et al., 2019 | Netherland | Hospitals and community | Seven personalized telephone counseling sessions over a 3-month period | IR | 0.042 | 11 |
| 60 | Stein-Seroussi et al., 2009 | US | Schools | ACTION program was based on stages of change theory, psychosocial theory, and the curriculum designers' qualitative research. The intervention included interactive games to teach cessation and abstinence skills. | CR | N/A, OR: 3.68, 95% CI: 0.83-16.39 | 9 |
| 61 | Thompson et al., 2007 | UK | Community nurse-led smoking cessation clinic | Group discussions that included discussing the association between smoking and health, information on smoking cessation, and advice on attending social events and how to manage passive smoking | QE/BA | N/A, t = 7.13 (df: 31), p < 0.001 | 11 |
| 62 | Vaid et al., 2014 | US | Community | On-line training session for clinic staff that took 45 to 60 minutes and covered guidelines and the complexity of smoking behaviors | QE/BA | 0.130 | 10 |
| 63 | Vartiainen et al., 2007 | Finland | Secondary school | Fourteen lessons about smoking and refusal skills training, provided over a 3-year period (5 lessons in the first/second year, and 4 lessons in the third year) | CR | 0.521 | 7 |
| 64 | Velasquez et al., 2017 | US | Primary care clinics | Two 40-minute motivational interviewing sessions provided by behavioral health specialists | IR | 0.110 | 10 |
| 65 | Vial et al., 2002 | Australia | The medical and surgical units at a Hospital | Nicotine patches provided by the research pharmacist, with follow-up one week after discharge from hospital | IR | 0.029 | 10 |
| 66 | WA et al., 1997 | US | City (middle-lower income area) | Community mobilization, direct educational activities, and a mass media campaign | IR | 0.031 | 8 |
| 67 | Wadland et al., 1999 | US | Low income and Medicaid covered community | A recruitment session followed by 6 computer-assisted counseling sessions provided by nurses and telephone counselors | QE | 0.010 | 11 |
| 68 | Wagner et al., 2016 | US | Poor and underserved urban community | The American Cancer Society FreshStart 4-week tobacco cessation program curriculum with examples of each of the topics | CR | 0.088 | 10 |
| 69 | Wewers et al., 2017 | US | Higher smoking prevalence counties | Seven face-to-face visits by community health workers | CR | 0.026 | 6 |
| 70 | Woodruff et al., 2007 | US | School | Seven, 45-minute, weekly sessions of internet-based, virtual reality, motivational interviewing by smoking cessation counselors | CR | 0.027 | 9 |
| 71 | Wu et al., 2003 | US | Low-income town | Twenty-minute video and role-play vignettes by 2 instructors | CR | 0.030 | 11 |
| 72 | Xiangyang et al., 2003 | China | Universities | Providing a series of lectures on smoking control, booklets and consultation about smoking cessation, setting up a non-smoking day, establishing student volunteer group, training teachers, opening a consulting hotline about smoking cessation to university students | QE/BA | 0.224 | 11 |
| 73 | Apata et al., 2019 | US | Low-income inner-city | Participants provided eight sessions, achieved milestones, joined the CEASE (Communities Engaged and Advocating for a Smoke-Free Environment) Partnership and referred other smokers to the program | QE /CT | 0.062 | 13 |
| 74 | Joshi et al., 2019 | India | Rural areas | Risk-reduction advice by community health workers through 6 household visits | IR | 0.025 | 11 |
| 75 | Khetan et al., 2018 | India | Semi-urban town with agriculture-based economy | Behavioral change communication through regular home adults and visits from community health workers | IR | −0.011 | 11 |
| 76 | Jiang et al., 2019 | Vietnam | Rural region | Advice and assistance from a health care provider advice and counseling by a village health worker | QE /CT | 0.152 | 9 |
| 77 | Snaterseet al., 2019 | Netherland | Communities | Usual care including visits to a cardiologist, cardiac rehabilitation, and up to four visits to a nurse-coordinated secondary prevention program; a risk status assessment; discussion about the current risk status with patients, assessment of the level of motivation for the current cardiovascular risk status, and official referral to the lifestyle program | QE /CT | 0.042 | 12 |
| 78 | Gilbodyet al., 2019 | UK | Hospitals and communities | Behavioral support from a mental health smoking cessation practitioner and pharmacological aids for smoking cessation | IR | 0.012 | 12 |
| 79 | Secades-Villa et al., 2019 | Spain | Communities | Therapy sessions (nicotine fading), and cognitive-behavioral treatment; information about tobacco, behavioral contraction, self-monitoring, graphical representation of cigarette smoking, stimulus control, strategies for controlling nicotine withdrawal symptoms, physical feedback consumption, training in alternative behaviors, and contingency management for shaping cessation period | IR | 0.042 | 11 |
| 80 | Bottorffet al., 2019 | Canada | Communities | Information and advice on practical strategies for quitting smoking, and information about the free nicotine replacement products and prescription for smoking cessation | QE/BA | 0.524 | 7 |

References 10 and 11 reported in the same studys and had reported similar effect sizes, so we used the effect size from Reference 11, the more recently published of the 2 references, in the analysis. References 54 and 57 reported on the same study and reported similar effect sizes, so we used the effect size from Reference 57, the more recently published of the 2 references, in the analysis.

BA: before-after study, CR: cluster randomized study, CT: controlled study, IR: individual randomized study, N/A: not available, QE: quasi-experimental study
