# Supplementary material for: Reporting Guidelines for Community-Based Participatory Research Did Not Improve the Reporting Quality of Published Studies: A Systematic Review of Studies on Smoking Cessation
Source: Int J Environ Res Public Health. 2020 May 31;17(11):3898. doi: 10.3390/ijerph17113898 (PMC7312250; doi:10.3390/ijerph17113898)
Supplement: Supplementary file 1 [file ijerph-17-03898-s001.zip › S7_Table_fin.docx]

**S7 Table.** **Eligibility criteria for the items in the checklist.**

| Item | Content |
| --- | --- |
|  | **Plan ahead for organizational structure** |
| q1 | The article has an Introduction, Method, and Result section or its equivalent structure, or  The article is organized by a hybrid of the above conventional headings with descriptive subheadings, or  The article is written about an introduction to the problem in the community and a rationale for their project, with conceptual and descriptive elements into a chronological framework. |
|  | **Convey the key elements of the project** |
| q2.1 | There is a description that the study was conducted in the region to solve the health problem which the region encountered. |
| q2.2 | There are specific dates and durations for how the project progressed. |
| q2.3 | There is a mention about the participants and/or co-researchers of the project. Exclude them if they are only listed as targets for intervention. |
| q2.4 | Their roles are specifically described. |
| q2.5 | The Method section of the paper mentions about the process and/or the methodology of the project. |
| q2.6 | The Result section of the paper describes the project outcomes and/or future actions. |
| q2.7 | Future research objectives are represented, or  The project finished. |
| Q2.8 | Charts, timelines, tables, or other graphics are used to describe project design. |
|  | **Convey the experiences of co-researchers** |
| q3.1 | The author's information includes information on the institution to which researchers belong and their qualifications, and  The Author contribution section lists who wrote the article. |
| q3.2 | In the paper, for example, in the Acknowledgement section, those, who participated in the study but did not write the paper, are described. |
| q3.3 | The words of an individual are specifically quoted, or  Personal opinions are aggregated, integrated, and expressed |
|  | **Address the challenges, pitfalls, and limitations of the project^*^** |
| q4.1 | The challenges, pitfalls, and limitations of the project are described. |
| q4.2 | How they were resolved through CBPR research is described, or  It is well documented that limitation is not fatal on the results of the study. |

^*^ We did not use the “What can we learn?” question in this review.
